# (*E*)-[({[(3-Methyl­phen­yl)meth­yl]sulfan­yl}methane­thio­yl)amino](1-phenyl­pentyl­idene)amine

**DOI:** 10.1107/S1600536811016965

**Published:** 2011-05-11

**Authors:** Georgiana Paulus, Karen A. Crouse, Mohamed Ibrahim Mohamed Tahir, Edward R. T. Tiekink

**Affiliations:** a Department of Chemistry, Universiti Putra Malaysia, 43400 Serdang, Malaysia; b Department of Chemistry, University of Malaya, 50603 Kuala Lumpur, Malaysia

## Abstract

In the structure of the title compound, C_20_H_24_N_2_S_2_, the central CN_2_S_2_ atoms are planar (r.m.s. deviation = 0.0205 Å) but both benzene rings are twisted out of this plane forming dihedral angles of 23.03 (6) and 84.75 (4)° (tol­yl); the *n*-butyl group occupies a position normal to the plane [N—C—C—C torsion angle = −84.33 (16)°]. The conformation of the imine bond [1.2888 (18) Å] is *E*. The *syn* arrangement of the thione S and amino H atoms enables the formation of N—H⋯S hydrogen bonds between centrosymmetrically related mol­ecules. These lead to eight-membered {⋯HNC=S}_2_ synthons which are further stabilized by proximate C—H⋯S interactions. The resulting dimeric aggregates are connected into a supra­molecular chain along the *c* axis by C—H⋯π(tol­yl) inter­actions.

## Related literature

For background on the coordination chemistry of hydrazine­carbodithio­ates, see: Ravoof *et al.* (2010 ▶). For related structures, see: Khoo *et al.* (2005 ▶); How *et al.* (2007 ▶).
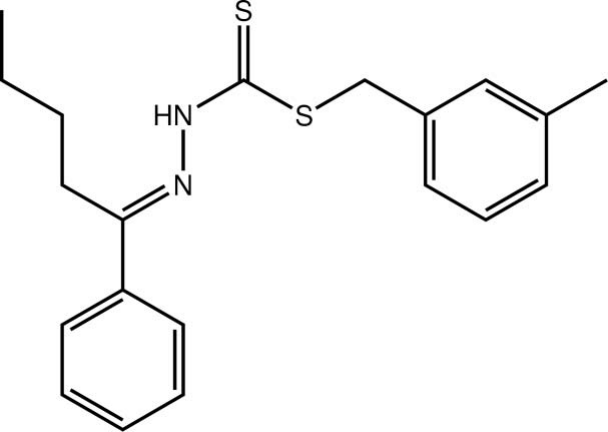


## Experimental

### 

#### Crystal data

C_20_H_24_N_2_S_2_
*M* *_r_* = 356.53
Monoclinic, 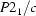

*a* = 11.3345 (1) Å
*b* = 19.1439 (3) Å
*c* = 8.6779 (1) Å
β = 95.802 (1)°
*V* = 1873.34 (4) Å^3^
*Z* = 4
Cu *K*α radiation
μ = 2.58 mm^−1^
*T* = 150 K
0.18 × 0.14 × 0.06 mm

#### Data collection

Oxford Diffraction Xcaliber Eos Gemini diffractometer
Absorption correction: multi-scan (*CrysAlis PRO*; Oxford Diffraction, 2010 ▶) *T*_min_ = 0.820, *T*_max_ = 0.924
33884 measured reflections
3631 independent reflections
3380 reflections with *I* > 2σ(*I*)
*R*_int_ = 0.023

#### Refinement

*R*[*F* ^2^ > 2σ(*F* ^2^)] = 0.033
*wR*(*F* ^2^) = 0.092
*S* = 1.03
3631 reflections
222 parameters
1 restraint
H atoms treated by a mixture of independent and constrained refinement
Δρ_max_ = 0.37 e Å^−3^
Δρ_min_ = −0.18 e Å^−3^

### 

Data collection: *CrysAlis PRO* (Oxford Diffraction, 2010 ▶); cell refinement: *CrysAlis PRO*; data reduction: *CrysAlis PRO*; program(s) used to solve structure: *SHELXS97* (Sheldrick, 2008 ▶); program(s) used to refine structure: *SHELXL97* (Sheldrick, 2008 ▶); molecular graphics: *ORTEP-3* (Farrugia, 1997 ▶) and *DIAMOND* (Brandenburg, 2006 ▶); software used to prepare material for publication: *publCIF* (Westrip, 2010 ▶).

## Supplementary Material

Crystal structure: contains datablocks global, I. DOI: 10.1107/S1600536811016965/hg5033sup1.cif

Structure factors: contains datablocks I. DOI: 10.1107/S1600536811016965/hg5033Isup2.hkl

Supplementary material file. DOI: 10.1107/S1600536811016965/hg5033Isup3.cml

Additional supplementary materials:  crystallographic information; 3D view; checkCIF report

## Figures and Tables

**Table 1:** Hydrogen-bond geometry (Å, °) *Cg*1 is the centroid of the C14–C19 ring.

*D*—H⋯*A*	*D*—H	H⋯*A*	*D*⋯*A*	*D*—H⋯*A*
N1—H1*N*⋯S1^i^	0.87 (1)	2.64 (1)	3.4926 (12)	165 (1)
C9—H9*B*⋯S1^i^	0.99	2.77	3.7576 (14)	178
C12—H12*C*⋯*Cg*1^ii^	0.98	2.81	3.7162 (14)	155

Symmetry codes: (i) 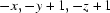; (ii) 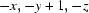.

**Fig. 1.**
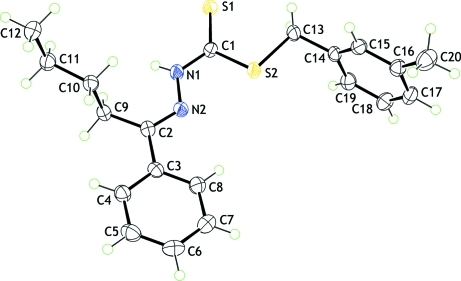
The molecular structure of of (I) showing the atom-labelling scheme and displacement ellipsoids at the 50% probability level.

**Fig. 2.**
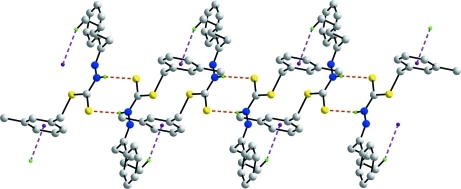
A view of the supramolecular chain in (I) mediated by N—H···S hydrogen bonding and C—H···π interactions, shown as orange and purple dashed lines, respectively.

**Fig. 3.**
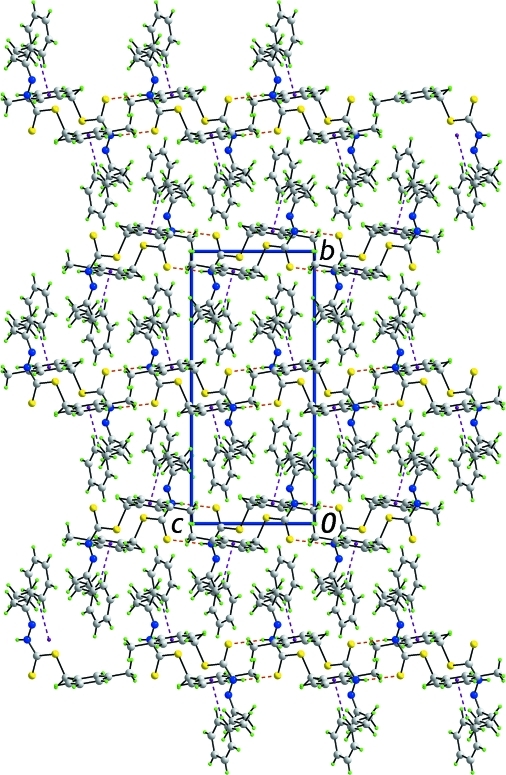
A view in projection down the a axis of the unit-cell contents for (I) showing inter-digitation of organic residues along the b axis. The N—H···S hydrogen bonding and C—H···π interactions are shown as orange and purple dashed lines, respectively.

## supplementary crystallographic information

### Comment

In continuing interest in the coordination chemistry of hydrazinecarbodithioate derivatives (Ravoof *et al.*, 2010), structural studies on the precursor molecules have been undertaken (Khoo *et al.* 2005; How *et al.* 2007). In this connection, the title compound, (I), the product of the condensation reaction between 3-methylbenzyldithiocarbazate and valerophenone, was investigated.

The central CN_2_S_2_ atoms in the molecular structure of (I), Fig. 1, are planar with a r.m.s. = 0.0205 Å. The adjacent residues are twisted out of this plane as seen in the values of the C1—N1—N2—C2 and C1—S2—C13—C14 torsion angles of 167.00 (12) and -163.64 (10) °, respectively. Further, the dihedral angles formed between the C3···C6 and C14···C19 benzene rings with the central plane are 23.03 (6) and 84.75 (4) °, respectively, indicating approximate co-planar and a perpendicular dispositions, respectively. Thus, the benzene rings are almost normal to each other, forming a dihedral angle between their respective least-squares planes of 80.13 (5) °. Finally, the *n*-butyl group, having an extended conformation, occupies a position approximately normal to the central plane with N2—C2—C9—C10 torsion angle being -84.33 (16) °. The conformation about the N2═C2 bond [1.2888 (18) Å] is *E*. The thione-S1 and amino-H atoms are *syn*, a disposition that allows for the formation of N—H···S hydrogen bonds between centrosymmetrically related molecules, Table 1. These lead to eight-membered {···HNC═S}_2_ synthons which are further stabilized by proximate C—H···S interactions, Table 1. The dimeric aggregates are connected into linear supramolecular chains along the *c* axis *via* C—H···π(C14···C19) contacts, Table 1 and Fig. 2. The chains pack into layers in the *ac* plane and their organic residues inter-digitate along the *b* axis, Fig. 3.

### Experimental

The precursor molecule 3-methylbenzyldithiocarbazate was prepared as previously described (Ravoof *et al.*, 2010). This (2.12 g, 0.01 mol) was dissolved in acetonitrile (35 ml) and valerophenone (1.62 g, 0.01 mol) was added. The temperature of the reaction mixture was maintained between 333–338 K with stirring over 30 min. as a yellow product formed. The product was filtered off, recrystallized and dried *in vacuo* over silica gel (yield 70%; *M*.pt. 366 K). Anal. Found (Calc.): C, 66.25 (67.37), H, 6.50 (6.78), N, 7.81 (7.86), S, 18.17 (17.99) %. Light-yellow crystals were grown from its acetonitrile solution through slow evaporation.

### Refinement

Carbon-bound H-atoms were placed in calculated positions (C—H 0.93 to 0.97 Å) and were included in the refinement in the riding model approximation, with *U*_iso_(H) set to 1.2 to 1.5*U*_equiv_(C). The amino H-atom was refined with a distance restraint of N—H = 0.88±0.01 Å with *U*_iso_(H) = 1.2*U*_eq_(N).

### Crystal data

**Table:**

C_20_H_24_N_2_S_2_	*F*(000) = 760
*M**_r_* = 356.53	*D*_x_ = 1.264 Mg m^−^^3^
Monoclinic, *P*2_1_/*c*	Cu *K*α radiation, λ = 1.54180 Å
Hall symbol: -P 2ybc	Cell parameters from 23382 reflections
*a* = 11.3345 (1) Å	θ = 3.9–71.2°
*b* = 19.1439 (3) Å	µ = 2.58 mm^−^^1^
*c* = 8.6779 (1) Å	*T* = 150 K
β = 95.802 (1)°	Block, pale-yellow
*V* = 1873.34 (4) Å^3^	0.18 × 0.14 × 0.06 mm
*Z* = 4	

### Data collection

**Table:**

Oxford Diffraction Xcaliber Eos Gemini diffractometer	3631 independent reflections
Radiation source: fine-focus sealed tube	3380 reflections with *I* > 2σ(*I*)
graphite	*R*_int_ = 0.023
Detector resolution: 16.1952 pixels mm^-1^	θ_max_ = 71.4°, θ_min_ = 3.9°
ω/2θ scans	*h* = −13→13
Absorption correction: multi-scan (*CrysAlis PRO*; Oxford Diffraction, 2010)	*k* = −22→23
*T*_min_ = 0.820, *T*_max_ = 0.924	*l* = −10→10
33884 measured reflections	

### Refinement

**Table:**

Refinement on *F*^2^	Primary atom site location: structure-invariant direct methods
Least-squares matrix: full	Secondary atom site location: difference Fourier map
*R*[*F*^2^ > 2σ(*F*^2^)] = 0.033	Hydrogen site location: inferred from neighbouring sites
*wR*(*F*^2^) = 0.092	H atoms treated by a mixture of independent and constrained refinement
*S* = 1.03	*w* = 1/[σ^2^(*F*_o_^2^) + (0.0606*P*)^2^ + 0.5891*P*] where *P* = (*F*_o_^2^ + 2*F*_c_^2^)/3
3631 reflections	(Δ/σ)_max_ = 0.002
222 parameters	Δρ_max_ = 0.37 e Å^−^^3^
1 restraint	Δρ_min_ = −0.18 e Å^−^^3^

### Special details

**Table:**

Geometry. All s.u.'s (except the s.u. in the dihedral angle between two l.s. planes) are estimated using the full covariance matrix. The cell s.u.'s are taken into account individually in the estimation of s.u.'s in distances, angles and torsion angles; correlations between s.u.'s in cell parameters are only used when they are defined by crystal symmetry. An approximate (isotropic) treatment of cell s.u.'s is used for estimating s.u.'s involving l.s. planes.
Refinement. Refinement of *F*^2^ against ALL reflections. The weighted *R*-factor *wR* and goodness of fit *S* are based on *F*^2^, conventional *R*-factors *R* are based on *F*, with *F* set to zero for negative *F*^2^. The threshold expression of *F*^2^ > 2σ(*F*^2^) is used only for calculating *R*-factors(gt) *etc*. and is not relevant to the choice of reflections for refinement. *R*-factors based on *F*^2^ are statistically about twice as large as those based on *F*, and *R*- factors based on ALL data will be even larger.

### Fractional atomic coordinates and isotropic or equivalent isotropic displacement parameters (Å^2^)

**Table:**

	*x*	*y*	*z*	*U*_iso_*/*U*_eq_	
S1	0.02007 (3)	0.442478 (17)	0.29961 (4)	0.02821 (11)	
S2	0.18648 (3)	0.521432 (17)	0.10978 (4)	0.02677 (11)	
N1	0.08701 (10)	0.57396 (6)	0.33862 (13)	0.0265 (3)	
H1N	0.0476 (14)	0.5734 (9)	0.4199 (15)	0.032*	
N2	0.15725 (10)	0.62898 (6)	0.29997 (13)	0.0257 (2)	
C1	0.09447 (11)	0.51415 (7)	0.25919 (15)	0.0233 (3)	
C2	0.13749 (12)	0.69067 (7)	0.35107 (15)	0.0239 (3)	
C3	0.21717 (12)	0.74591 (7)	0.29942 (15)	0.0245 (3)	
C4	0.19755 (13)	0.81656 (8)	0.32641 (17)	0.0298 (3)	
H4	0.1342	0.8301	0.3837	0.036*	
C5	0.26964 (14)	0.86719 (8)	0.27053 (19)	0.0357 (3)	
H5	0.2548	0.9152	0.2888	0.043*	
C6	0.36287 (14)	0.84832 (9)	0.18845 (19)	0.0368 (3)	
H6	0.4120	0.8832	0.1503	0.044*	
C7	0.38448 (13)	0.77831 (9)	0.16191 (19)	0.0362 (3)	
H7	0.4489	0.7651	0.1062	0.043*	
C8	0.31210 (13)	0.72760 (8)	0.21670 (17)	0.0306 (3)	
H8	0.3272	0.6797	0.1978	0.037*	
C9	0.03642 (12)	0.70935 (7)	0.44343 (15)	0.0253 (3)	
H9A	0.0578	0.7511	0.5075	0.030*	
H9B	0.0215	0.6703	0.5137	0.030*	
C10	−0.07651 (12)	0.72428 (7)	0.33483 (15)	0.0265 (3)	
H10A	−0.0635	0.7664	0.2724	0.032*	
H10B	−0.0918	0.6845	0.2627	0.032*	
C11	−0.18508 (13)	0.73576 (8)	0.42165 (17)	0.0308 (3)	
H11A	−0.1981	0.6936	0.4839	0.037*	
H11B	−0.1696	0.7754	0.4941	0.037*	
C12	−0.29719 (13)	0.75075 (9)	0.3150 (2)	0.0372 (3)	
H12A	−0.2865	0.7937	0.2567	0.056*	
H12B	−0.3642	0.7565	0.3770	0.056*	
H12C	−0.3131	0.7117	0.2428	0.056*	
C13	0.17797 (13)	0.43434 (7)	0.02445 (18)	0.0296 (3)	
H13A	0.1842	0.3982	0.1064	0.035*	
H13B	0.1018	0.4281	−0.0407	0.035*	
C14	0.28092 (12)	0.42893 (7)	−0.07279 (16)	0.0260 (3)	
C15	0.26557 (12)	0.44105 (7)	−0.23135 (17)	0.0269 (3)	
H15	0.1886	0.4515	−0.2797	0.032*	
C16	0.36119 (13)	0.43818 (7)	−0.32117 (17)	0.0285 (3)	
C17	0.47312 (13)	0.42296 (8)	−0.24810 (17)	0.0310 (3)	
H17	0.5392	0.4209	−0.3072	0.037*	
C18	0.48925 (13)	0.41079 (8)	−0.08963 (18)	0.0333 (3)	
H18	0.5661	0.4004	−0.0411	0.040*	
C19	0.39395 (13)	0.41375 (8)	−0.00206 (17)	0.0309 (3)	
H19	0.4055	0.4054	0.1063	0.037*	
C20	0.34264 (15)	0.45187 (10)	−0.49302 (19)	0.0431 (4)	
H20A	0.3238	0.5013	−0.5112	0.065*	
H20B	0.2770	0.4231	−0.5395	0.065*	
H20C	0.4151	0.4401	−0.5401	0.065*	

### Atomic displacement parameters (Å^2^)

**Table:**

	*U*^11^	*U*^22^	*U*^33^	*U*^12^	*U*^13^	*U*^23^
S1	0.0308 (2)	0.02422 (19)	0.03141 (19)	−0.00527 (12)	0.01214 (14)	−0.00176 (13)
S2	0.03181 (19)	0.02038 (18)	0.03037 (19)	−0.00134 (12)	0.01409 (14)	−0.00156 (12)
N1	0.0300 (6)	0.0232 (6)	0.0281 (6)	−0.0013 (4)	0.0118 (5)	−0.0015 (4)
N2	0.0273 (6)	0.0215 (6)	0.0290 (6)	−0.0007 (4)	0.0066 (4)	−0.0008 (4)
C1	0.0221 (6)	0.0231 (6)	0.0252 (6)	0.0019 (5)	0.0049 (5)	0.0017 (5)
C2	0.0262 (6)	0.0242 (7)	0.0211 (6)	0.0035 (5)	0.0008 (5)	0.0000 (5)
C3	0.0254 (6)	0.0239 (7)	0.0233 (6)	0.0009 (5)	−0.0019 (5)	−0.0007 (5)
C4	0.0299 (7)	0.0257 (7)	0.0334 (7)	0.0020 (5)	0.0019 (6)	−0.0028 (6)
C5	0.0381 (8)	0.0242 (7)	0.0438 (8)	−0.0020 (6)	−0.0003 (7)	−0.0010 (6)
C6	0.0346 (8)	0.0354 (8)	0.0400 (8)	−0.0110 (6)	0.0016 (6)	0.0045 (6)
C7	0.0295 (7)	0.0403 (9)	0.0399 (8)	−0.0018 (6)	0.0088 (6)	0.0002 (7)
C8	0.0296 (7)	0.0273 (7)	0.0353 (7)	0.0017 (6)	0.0052 (6)	−0.0011 (6)
C9	0.0304 (7)	0.0224 (6)	0.0237 (6)	0.0014 (5)	0.0052 (5)	−0.0014 (5)
C10	0.0275 (7)	0.0265 (7)	0.0260 (6)	−0.0003 (5)	0.0050 (5)	−0.0018 (5)
C11	0.0312 (7)	0.0293 (7)	0.0332 (7)	0.0011 (6)	0.0092 (6)	−0.0005 (6)
C12	0.0287 (7)	0.0371 (8)	0.0466 (9)	0.0038 (6)	0.0077 (6)	−0.0051 (7)
C13	0.0322 (7)	0.0217 (7)	0.0370 (7)	−0.0021 (5)	0.0137 (6)	−0.0064 (6)
C14	0.0288 (7)	0.0184 (6)	0.0323 (7)	−0.0002 (5)	0.0107 (5)	−0.0039 (5)
C15	0.0249 (7)	0.0216 (7)	0.0346 (7)	0.0012 (5)	0.0050 (5)	0.0001 (5)
C16	0.0298 (7)	0.0259 (7)	0.0306 (7)	−0.0002 (5)	0.0069 (6)	0.0012 (5)
C17	0.0266 (7)	0.0327 (7)	0.0353 (7)	0.0032 (6)	0.0116 (6)	0.0011 (6)
C18	0.0256 (7)	0.0369 (8)	0.0374 (8)	0.0052 (6)	0.0035 (6)	0.0026 (6)
C19	0.0340 (7)	0.0311 (8)	0.0282 (7)	0.0014 (6)	0.0065 (6)	0.0000 (6)
C20	0.0372 (9)	0.0600 (11)	0.0326 (8)	−0.0027 (8)	0.0063 (7)	0.0077 (7)

### Geometric parameters (Å, °)

**Table:**

S1—C1	1.6663 (13)	C10—H10B	0.9900
S2—C1	1.7497 (13)	C11—C12	1.522 (2)
S2—C13	1.8229 (14)	C11—H11A	0.9900
N1—C1	1.3434 (18)	C11—H11B	0.9900
N1—N2	1.3820 (16)	C12—H12A	0.9800
N1—H1N	0.872 (9)	C12—H12B	0.9800
N2—C2	1.2888 (18)	C12—H12C	0.9800
C2—C3	1.4889 (19)	C13—C14	1.5111 (18)
C2—C9	1.5061 (18)	C13—H13A	0.9900
C3—C4	1.394 (2)	C13—H13B	0.9900
C3—C8	1.397 (2)	C14—C15	1.389 (2)
C4—C5	1.387 (2)	C14—C19	1.394 (2)
C4—H4	0.9500	C15—C16	1.399 (2)
C5—C6	1.381 (2)	C15—H15	0.9500
C5—H5	0.9500	C16—C17	1.391 (2)
C6—C7	1.386 (2)	C16—C20	1.508 (2)
C6—H6	0.9500	C17—C18	1.388 (2)
C7—C8	1.386 (2)	C17—H17	0.9500
C7—H7	0.9500	C18—C19	1.383 (2)
C8—H8	0.9500	C18—H18	0.9500
C9—C10	1.5377 (19)	C19—H19	0.9500
C9—H9A	0.9900	C20—H20A	0.9800
C9—H9B	0.9900	C20—H20B	0.9800
C10—C11	1.5224 (19)	C20—H20C	0.9800
C10—H10A	0.9900		
			
C1—S2—C13	102.54 (6)	C12—C11—H11A	108.9
C1—N1—N2	117.22 (11)	C10—C11—H11A	108.9
C1—N1—H1N	118.0 (12)	C12—C11—H11B	108.9
N2—N1—H1N	124.0 (12)	C10—C11—H11B	108.9
C2—N2—N1	119.37 (11)	H11A—C11—H11B	107.8
N1—C1—S1	122.30 (10)	C11—C12—H12A	109.5
N1—C1—S2	112.67 (10)	C11—C12—H12B	109.5
S1—C1—S2	125.01 (8)	H12A—C12—H12B	109.5
N2—C2—C3	114.55 (12)	C11—C12—H12C	109.5
N2—C2—C9	124.65 (12)	H12A—C12—H12C	109.5
C3—C2—C9	120.57 (11)	H12B—C12—H12C	109.5
C4—C3—C8	118.29 (13)	C14—C13—S2	106.10 (9)
C4—C3—C2	121.77 (12)	C14—C13—H13A	110.5
C8—C3—C2	119.90 (12)	S2—C13—H13A	110.5
C5—C4—C3	120.64 (14)	C14—C13—H13B	110.5
C5—C4—H4	119.7	S2—C13—H13B	110.5
C3—C4—H4	119.7	H13A—C13—H13B	108.7
C6—C5—C4	120.42 (14)	C15—C14—C19	119.24 (13)
C6—C5—H5	119.8	C15—C14—C13	121.02 (13)
C4—C5—H5	119.8	C19—C14—C13	119.71 (13)
C5—C6—C7	119.73 (14)	C14—C15—C16	121.27 (13)
C5—C6—H6	120.1	C14—C15—H15	119.4
C7—C6—H6	120.1	C16—C15—H15	119.4
C8—C7—C6	119.99 (14)	C17—C16—C15	118.48 (13)
C8—C7—H7	120.0	C17—C16—C20	121.12 (13)
C6—C7—H7	120.0	C15—C16—C20	120.40 (14)
C7—C8—C3	120.92 (14)	C18—C17—C16	120.63 (13)
C7—C8—H8	119.5	C18—C17—H17	119.7
C3—C8—H8	119.5	C16—C17—H17	119.7
C2—C9—C10	110.42 (11)	C19—C18—C17	120.35 (14)
C2—C9—H9A	109.6	C19—C18—H18	119.8
C10—C9—H9A	109.6	C17—C18—H18	119.8
C2—C9—H9B	109.6	C18—C19—C14	120.03 (13)
C10—C9—H9B	109.6	C18—C19—H19	120.0
H9A—C9—H9B	108.1	C14—C19—H19	120.0
C11—C10—C9	112.83 (11)	C16—C20—H20A	109.5
C11—C10—H10A	109.0	C16—C20—H20B	109.5
C9—C10—H10A	109.0	H20A—C20—H20B	109.5
C11—C10—H10B	109.0	C16—C20—H20C	109.5
C9—C10—H10B	109.0	H20A—C20—H20C	109.5
H10A—C10—H10B	107.8	H20B—C20—H20C	109.5
C12—C11—C10	113.17 (12)		
			
C1—N1—N2—C2	167.00 (12)	C2—C3—C8—C7	177.31 (13)
N2—N1—C1—S1	176.84 (10)	N2—C2—C9—C10	−84.33 (16)
N2—N1—C1—S2	−4.54 (15)	C3—C2—C9—C10	89.91 (14)
C13—S2—C1—N1	−179.56 (10)	C2—C9—C10—C11	173.76 (11)
C13—S2—C1—S1	−0.98 (11)	C9—C10—C11—C12	179.86 (12)
N1—N2—C2—C3	−178.88 (11)	C1—S2—C13—C14	−163.64 (10)
N1—N2—C2—C9	−4.3 (2)	S2—C13—C14—C15	−97.18 (14)
N2—C2—C3—C4	171.08 (12)	S2—C13—C14—C19	80.75 (14)
C9—C2—C3—C4	−3.72 (19)	C19—C14—C15—C16	0.1 (2)
N2—C2—C3—C8	−6.59 (18)	C13—C14—C15—C16	178.05 (12)
C9—C2—C3—C8	178.62 (12)	C14—C15—C16—C17	−0.1 (2)
C8—C3—C4—C5	0.9 (2)	C14—C15—C16—C20	−179.68 (14)
C2—C3—C4—C5	−176.83 (13)	C15—C16—C17—C18	0.1 (2)
C3—C4—C5—C6	−0.6 (2)	C20—C16—C17—C18	179.67 (15)
C4—C5—C6—C7	0.0 (2)	C16—C17—C18—C19	−0.1 (2)
C5—C6—C7—C8	0.5 (2)	C17—C18—C19—C14	0.1 (2)
C6—C7—C8—C3	−0.2 (2)	C15—C14—C19—C18	−0.1 (2)
C4—C3—C8—C7	−0.4 (2)	C13—C14—C19—C18	−178.05 (13)

### Hydrogen-bond geometry (Å, °)

**Table:**

*D*—H···*A*	*D*—H	H···*A*	*D*···*A*	*D*—H···*A*
N1—H1N···S1^i^	0.87 (1)	2.64 (1)	3.4926 (12)	165.(1)
C9—H9B···S1^i^	0.99	2.77	3.7576 (14)	178
C12—H12C···Cg1^ii^	0.98	2.81	3.7162 (14)	155

Symmetry codes: (i) −*x*, −*y*+1, −*z*+1; (ii) −*x*, −*y*+1, −*z*.
